# Comparison of Immuno-PET of CD138 and PET imaging with ^64^CuCl_2_ and ^18^F-FDG in a preclinical syngeneic model of multiple myeloma

**DOI:** 10.18632/oncotarget.23886

**Published:** 2018-01-03

**Authors:** Clément Bailly, Sébastien Gouard, Marie Lacombe, Patricia Remaud-Le Saëc, Benjamin Chalopin, Mickaël Bourgeois, Nicolas Chouin, Raphaël Tripier, Zakaria Halime, Ferid Haddad, Alain Faivre-Chauvet, Françoise Kraeber-Bodéré, Michel Chérel, Caroline Bodet-Milin

**Affiliations:** ^1^ Nuclear Medicine Department, University Hospital, Nantes, France; ^2^ Nantes-Angers Cancer Research Center CRCINA, University of Nantes, INSERM UMR1232, CNRS-ERL6001, Nantes, France; ^3^ Nuclear Medicine Department, ICO-René Gauducheau Cancer Center, Saint-Herblain, France; ^4^ Groupement d’Intérêt Public ARRONAX, Saint-Herblain, France; ^5^ AMaROC, Oniris, Ecole Nationale Vétérinaire, Agroalimentaire et de l'Alimentation de Nantes-Atlantique, Nantes, France; ^6^ CNRS-UMR6521, University of Bretagne Occidentale, Brest, France

**Keywords:** multiple myeloma, immuno-PET, copper-64, murine CD138, syngeneic model

## Abstract

**Purpose:**

Although recent data from the literature suggest that PET imaging with [18]-Fluorodeoxyglucose (^18^F-FDG) is a promising technique in multiple myeloma (MM), the development of other radiopharmaceuticals seems relevant. CD138 is currently used as a standard marker in many laboratories for the identification and purification of myeloma cells, and could be used in phenotype tumor imaging. In this study, we evaluated a ^64^Cu-labeled anti-CD138 murine antibody (^64^Cu-TE2A-9E7.4) and a metabolic tracer (^64^CuCl_2_) for PET imaging in a MM syngeneic mouse model.

**Experimental Design and Results:**

^64^Cu-TE2A-9E7.4 antibody and ^64^CuCl2 were evaluated via PET imaging and biodistribution studies in C57BL / KaLwRij mice bearing either 5T33-MM subcutaneous tumors or bone lesions. These results were compared to 18F-FDG-PET imaging. Autoradiography and histology of representative tumors were secondly conducted. In biodistribution and PET studies, ^64^Cu-TE2A-9E7.4 displayed good tumor uptake of subcutaneous and intra-medullary lesions, greater than that demonstrated with ^18^F-FDG-PET. In control experiments, only low-level, non-specific uptake of ^64^Cu-labeled isotype IgG was observed in tumors. Similarly, low activity concentrations of ^64^CuCl_2_ were accumulated in MM lesions. Histopathologic analysis of the immuno-PET–positive lesions revealed the presence of plasma cell infiltrates within the bone marrow.

**Conclusions:**

^64^Cu-labeled anti-CD138 antibody can detect subcutaneous MM tumors and bone marrow lesions with high sensitivity, outperforming ^18^F-FDG-PET and ^64^CuCl_2_ in this preclinical model. These data support ^64^Cu-anti-CD138 antibody as a specific and promising new imaging radiopharmaceutical agent in MM.

## INTRODUCTION

Multiple myeloma (MM) is a hematological neoplasm characterized by the clonal proliferation of malignant plasma cells in the bone marrow [1]. It is a rare disease that accounts for approximately 15% of hematological malignancies and results in a diffuse infiltration of the bone marrow, focal bone lesions and extra-medullary lesions. Over the past two decades, advances have been made with regard to the diagnosis, staging, treatment and imaging of MM [1, 2]. Magnetic resonance imaging (MRI) is currently recommended as the most effective imaging for MM at diagnosis [2]. Positron emission tomography (PET) combined with computed tomography using [18]Fluorodeoxyglucose (^18^F-FDG) is still being evaluated for initial staging and therapeutic monitoring in this pathology and its place needs to be validated [3, 4]. However, ^18^F-FDG being a non-tumor-specific metabolic tracer, development of phenotype tumor PET imaging is an attractive novel option to improve tumor characterization by targeting biomarkers expressed by neoplasm’s cells [5]. The combination of PET with monoclonal antibodies (mAbs), combining the high sensitivity and resolution of a PET camera with the specificity of a mAb allows the production of a specific imaging, called immuno-PET [6]. In MM, among the targeted antigens, CD138 or syndecan-1 is a cell surface proteoglycan that plays an important role in regulating cell signaling [7]. It is expressed by viable MM cells in the bone marrow and peripheral blood as well as differentiated plasma cells [8] and is currently used as a standard marker in many laboratories for the identification and purification of myeloma cells. CD138 PET imaging may thus specifically image MM lesions. Moreover, Phase I–II studies have been initiated with an antiCD138mAb [9, 10] and stronger response might be obtained by conjugating antiCD138mAb with radioisotopes, as previously demonstrated with radiolabeled antiCD20mAb in lymphoma [11]. Besides, based on the evidence that the human copper transporter 1 (CTR1) is overexpressed in neoplastic tissues, ^64^CuCl_2_ has been reported as a promising PET probe for imaging a variety of cancers such as prostate cancer and melanoma [12–14].

Here, we report preclinical PET imaging of CD138 in a subcutaneous and bone marrow disseminated mouse model of orthotopic syngeneic MM (C57BL / KaLwRij and 5T33 cells) using Copper-64 labeled mCD138-specific 9E7.4 antibody (^64^Cu-TE2A-9E7.4) compared to ^18^F-FDG-PET, bioluminescence imaging and ^64^CuCl_2_ imaging both as a control of potential copper release by the chelator agent and as a molecular imaging probe.

## RESULTS

### *Ex vivo* biodistribution experiments

**Figure 1:**
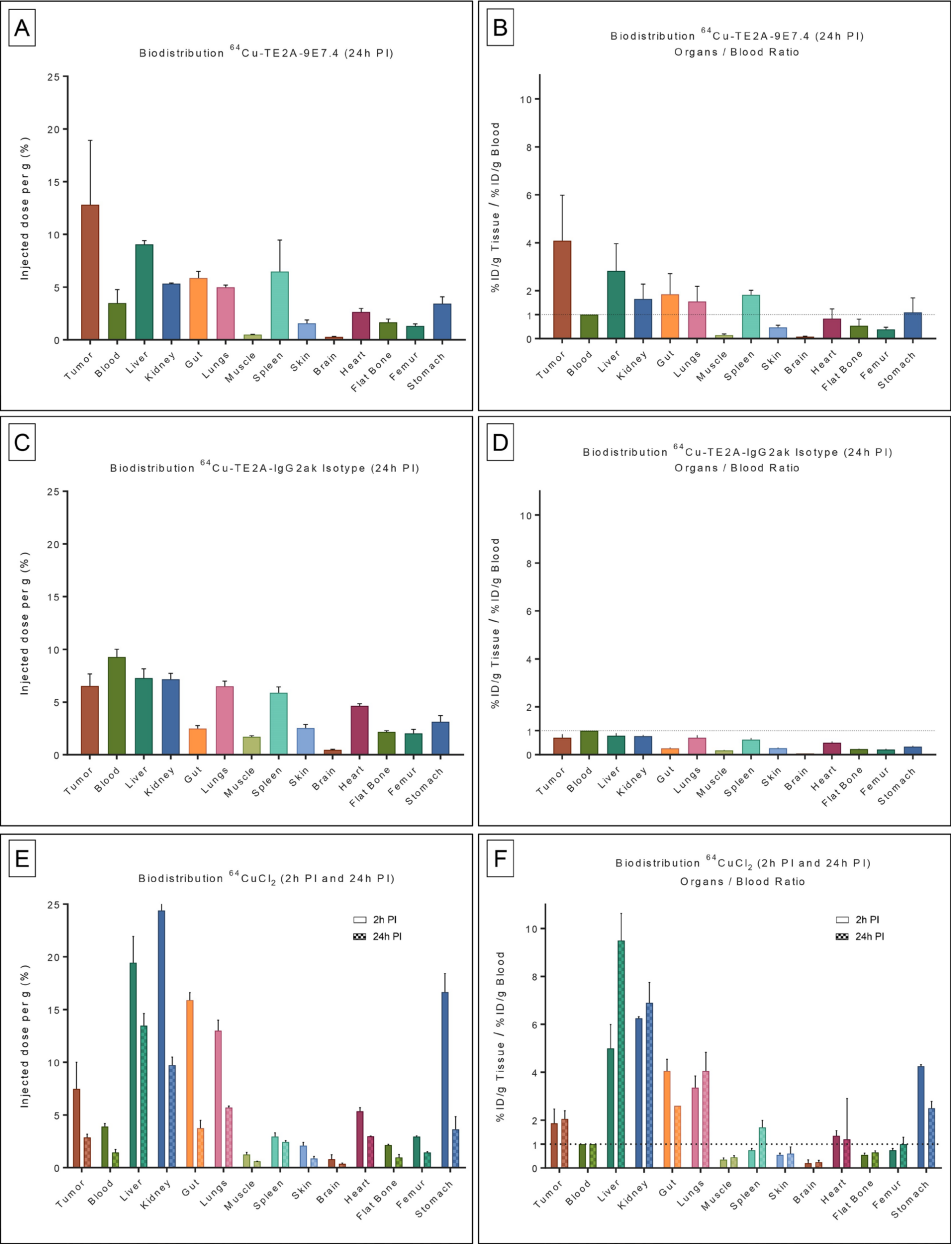
Biodistribution results and organ-to-blood ratios of ^64^Cu-TE2A-9E7.4, ^64^Cu-TE2A-IgG2a k Isotype and ^64^CuCl2 in tumor-bearing mice *Ex vivo* biodistribution results (**A**) and organ-to-blood ratios (**B**) of ^64^Cu-TE2A-9E7.4 at 24 h post-injection (PI), in the subcutaneous tumor model (*n* = 3). *Ex vivo* biodistribution results (**C**) and organ-to-blood ratios (**D**) of ^64^Cu-TE2A-IgG2a k Isotype at 24 h PI (*n* = 3). *Ex vivo* biodistribution results (**E**) and organ-to-blood ratios (**F**) of ^64^CuCl_2_ at 2 h and 24 h PI (*n* = 3 for each group). Values are expressed in percentage of the injected radioactive dose per gram of tissue (%ID/g) and presented as mean +/− SD.

*Ex vivo* biodistribution results are presented in Figure 1. On the study conducted 24 h after administration of ^64^Cu-TE2A-9E7.4 (Figure 1A and 1B) in a subcutaneous model of MM, the highest accumulation was observed in tumors compared to all other samples collected (12.82 ± 6.09% injected dose per gram [%ID/g] at 24 h post injection (PI)) with high tumor-to-blood ratios (4.08 ± 1.9 at 24 h PI). ^64^Cu-TE2A-9E7.4 displayed rapid blood clearance as determined by the radioactivity remaining in the blood at 24 h PI (3.47 ± 1.28% ID/g). The radioimmunoconjugate also showed low muscle uptake of 0.49 ± 0.03% ID/g at 24 h PI. Relative high uptakes of ^64^Cu-TE2A-9E7.4 was found in several normal organs such as liver (9.04 ± 0.36% ID/g at 24 h PI) and spleen (6.46 ± 2.99% ID/g at 24 h PI). All other organs displayed activity concentrations of 5%ID/g or less at 24 h PI. As a control of specificity of the ^64^Cu-TE2A-9E7.4, biodistribution experiments at 24 h PI of ^64^Cu-TE2A-IgG2ak Isotype was performed (Figure 1C and 1D). It showed persistent high activity in the blood (9.26 ± 0.75%ID/g at 24 h PI) and relative high uptakes in several normal organs including tumors (6.53 ± 1.14%ID/g at 24 h PI) resulting in very poor tumor-to-blood ratios (0.71 ± 0.15 at 24 h PI).

Biodistribution of ^64^CuCl_2_ was determined at 2 h and 24 h after injection (Figure 1E and 1F). ^64^CuCl_2_ displayed rapid yet modest accumulation in the tumors (7.47 ± 2.52% ID/g at 2 h PI) which slightly decreased over time (2.87 ± 0.32% ID/g at 24 h PI). ^64^CuCl_2_ showed significant blood clearance from 2 h PI (3.9 ± 0.28% ID/g) to 24h PI (1.43 ± 0.29% ID/g), resulting in stable tumor-to-blood ratios (1.88 ± 0.59 at 2 h PI and 2.05 ± 0.34 at 24 h PI). Relative high uptakes of ^64^CuCl_2_ was observed in non-target organs such as liver, kidney, lung, gut and stomach. Except for the liver (19.45 ± 2.47%ID/g at 2 h PI; 13.48 ± 1.13% ID/g at 24 h PI) and kidney (24.40 ± 2.12%ID/g at 2h PI; 9.73 ± 0.76% ID/g at 24h PI), these high uptakes clearly decreased at 24 h PI.

### PET imaging of subcutaneous tumor

**Figure 2:**
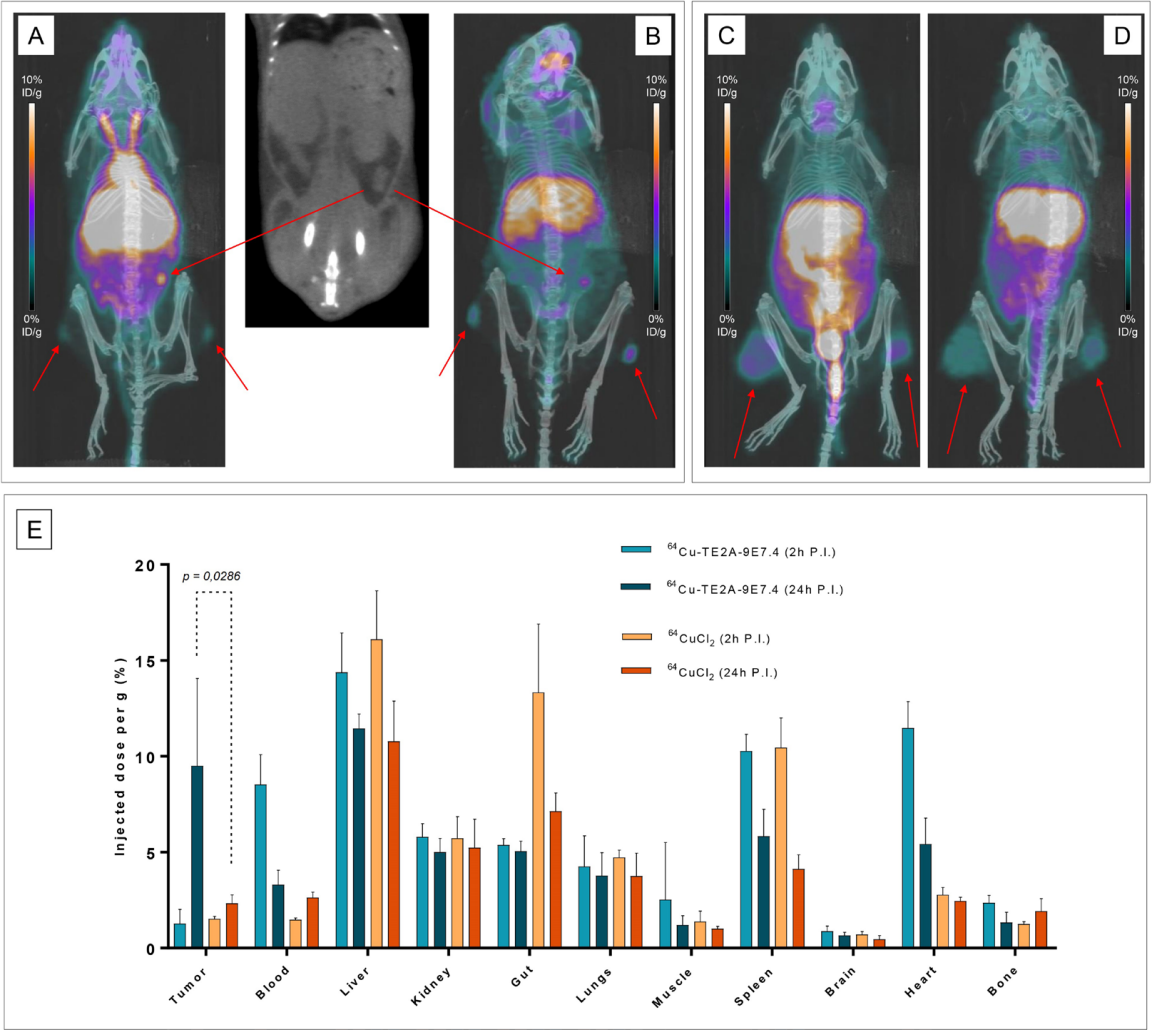
PET imaging and quantification with ^64^Cu-TE2A-9E7.4 and ^64^CuCl2 in tumor-bearing mice Maximum intensity projections of PET and CT imaging at 2 h post-injection (PI) (**A**) and at 24 h PI (**B**) of Mouse 2 showing uptakes in both subcutaneous tumors and of an iliac lymph node (Tumors are indicated by arrows). Maximum intensity projections of PET and CT imaging with ^64^CuCl_2_ at 2 h PI (**C**) and at 24 h PI (**D**) of Mouse 8 showing uptakes in both subcutaneous tumors. PET quantification of tumors and majors organs at 2 h and 24 h PI (**E**) of ^64^Cu-TE2A-9E7.4 and ^64^CuCl_2_ (*n* = 3 for each group). Values are expressed in percentage of the injected radioactive dose per gram of tissue (%ID/g) and presented as mean +/− SD. Comparison between quantification analysis of PET images obtained after 24 h PI of ^64^Cu-TE2A-9E7.4 and ^64^CuCl_2_ showed net higher uptake in tumors for the first probe (9.5 ± 4.5 vs 2.32 ± 0.45 respectively for ^64^Cu-TE2A-9E7.4 and ^64^CuCl_2_ at 24 h PI; *p* = 0.0286; non-parametric test).

PET imaging experiments (Figure 2) confirmed biodistribution observations and helped to visualize *in vivo* distributions of ^64^Cu-TE2A-9E7.4 and ^64^CuCl_2_ over time. Data plotted in Figure 2E were consistent with the biodistribution data (Figure 1). For ^64^Cu-TE2A-9E7.4, PET images illustrated the progressive selective targeting of SC tumors (and lymph node for Mouse 2), which increased from 2 h PI to 24 h PI while a concomitant decrease in blood and bone (predominant on the last lumbar vertebrae, the sacroiliac, coxo-femoral joints and knees) activity was observed (Figures 2A and 2B). Intense liver uptake and moderate to intense digestive uptake were also visible at 2 h PI, which decreased at 24 h PI.

On PET images obtained after ^64^CuCl_2_ injection, SC tumors were clearly visible at both times yet with modest accumulation (Figures 2C and 2D). Moreover radioactivity and contrast decreased between 2 h and 24 h PI. High liver, kidney and intestines uptakes were observed at all the time points.

Comparison between quantification analysis of PET images obtained after 24 h PI of ^64^Cu-TE2A-9E7.4 and ^64^CuCl_2_ showed net higher uptake in tumors for the first probe (9.5 ± 4.5 vs 2.32 ± 0.45 respectively for ^64^Cu-TE2A-9E7.4 and ^64^CuCl_2_ at 24 h PI; *p* = 0.0286; non-parametric test) (Figure 2E).

### Digital autoradiography

**Figure 3:**
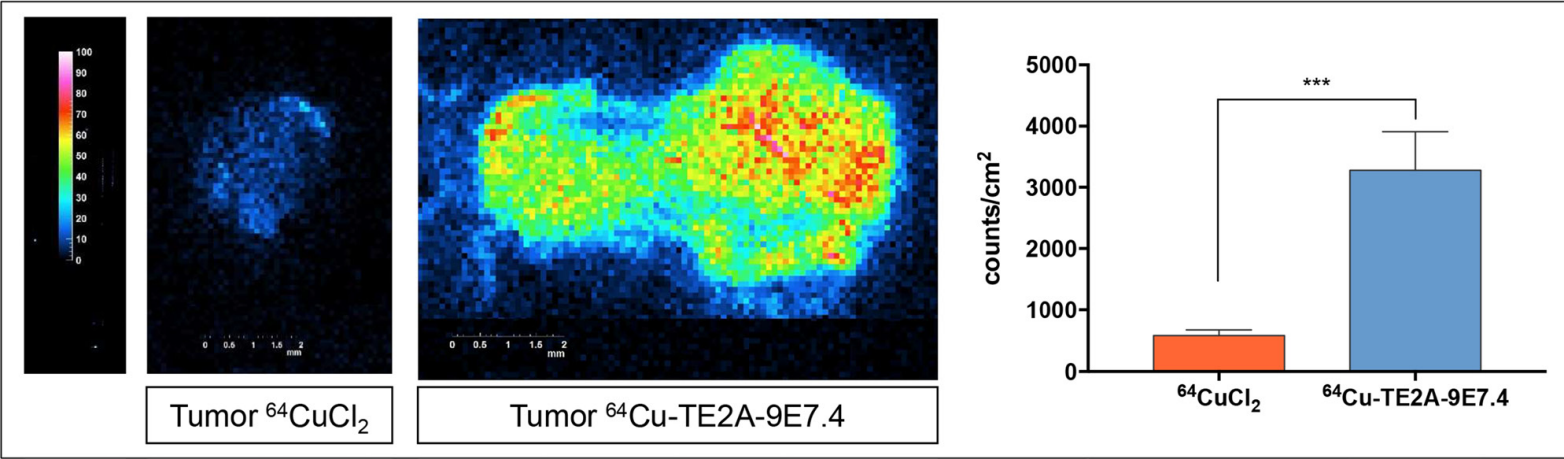
Digital autoradiography acquisitions performed on subcutaneous tumors of Mice 1 and 7, respectively imaged with ^64^Cu-TE2A-9E7.4 and ^64^CuCl2 Measurement of counts/mm^2^ found a significant difference (non-parametric test) between ^64^CuCl_2_ and ^64^Cu-TE2A-9E7.4 signals.

Digital autoradiography acquisitions were performed on SC tumors of Mice 1 and 7, respectively imaged with ^64^Cu-TE2A-9E7.4 and ^64^CuCl_2_ (Figure 3). Firstly, tumor section obtained with ^64^Cu-TE2A-9E7.4 revealed a high heterogeneity in the distribution of the vector. Secondly, measurement of counts/mm^2^ found a significant difference (583.3 ± 54.3 vs 3277 ± 316.6 respectively for ^64^CuCl_2_ and ^64^Cu-TE2A-9E7.4; *p* = 0.0008; non-parametric test) between ^64^CuCl_2_ and ^64^Cu-TE2A-9E7.4 signals reflecting the observed excellent uptake.

### PET imaging of disseminated disease

To establish a model of disseminated disease, mice were injected intravenously and the distribution was serially assessed using bioluminescence. Mice injected IV developed lesions in the skull, spine, sacrum and members (Supplementary Table 1).

**Figure 4:**
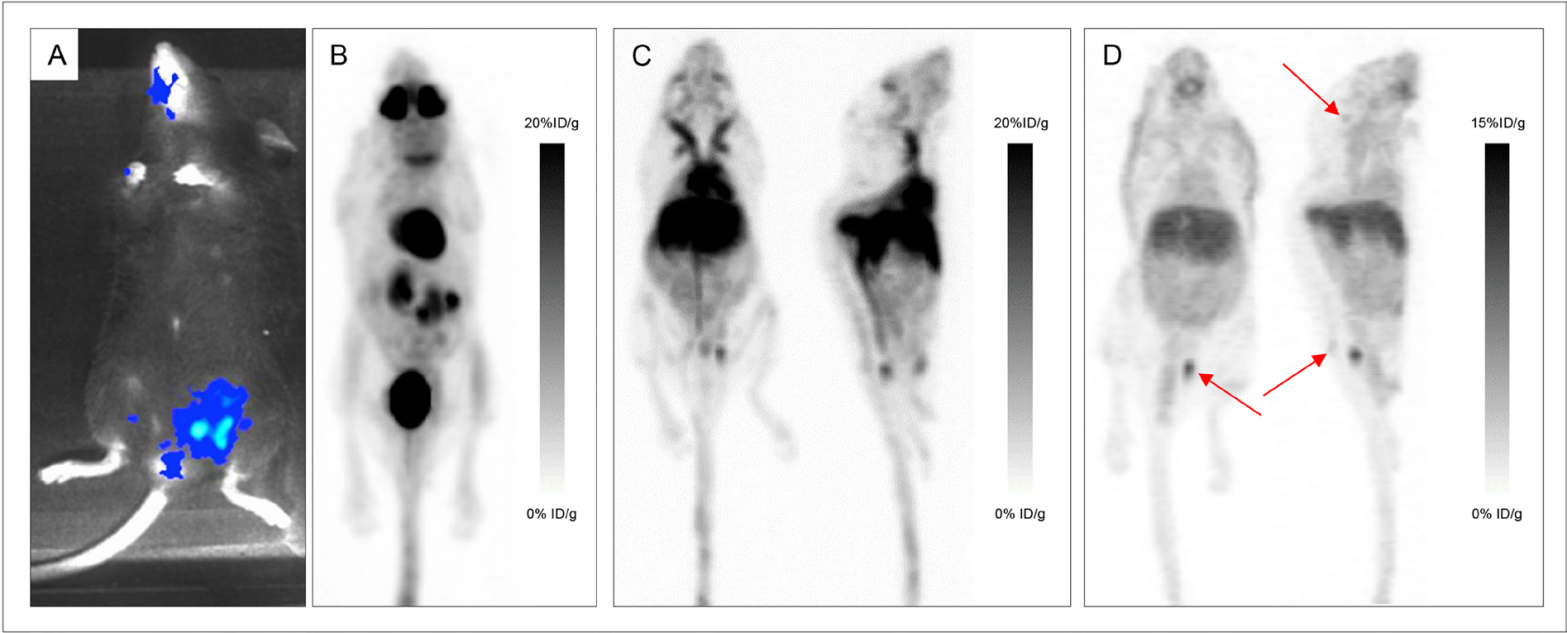
Various explorations conducted in Mouse 14 Bioluminescence imaging (**A**), maximum intensity projections of ^18^F-FDG-PET and CT imaging (**B**), maximum intensity projections of PET and CT imaging with ^64^Cu-TE2A-9E7.4 at 2h post-injection (PI) (**C**) and 24 h PI (**D**) showing uptakes in the skull, sacrum and left iliac wing (Tumors are indicated by arrows).

**Figure 5:**
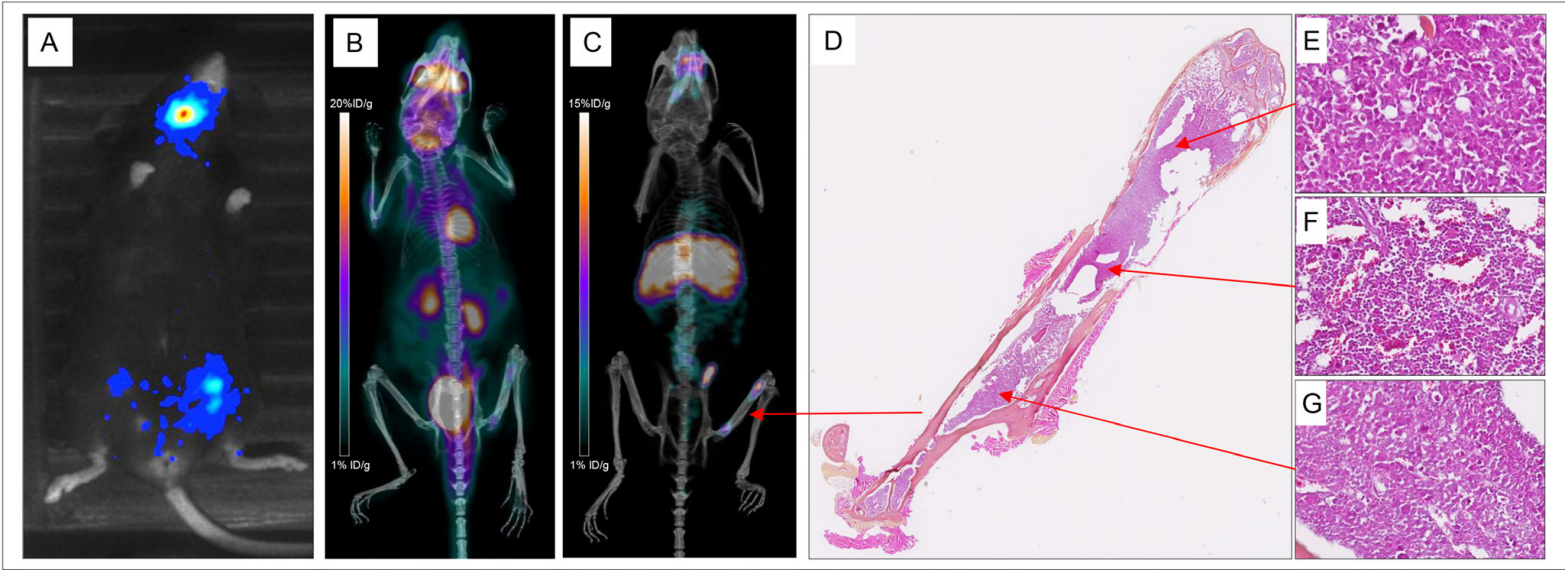
Various explorations conducted in Mouse 13 Bioluminescence imaging (**A**), maximum intensity projections of ^18^F-FDG-PET and CT (**B**), maximum intensity projections of PET and CT imaging with ^64^Cu-TE2A-9E7.4 at 24 h post-injection (**C**) and histological analysis of the left femur stained with hematoxylin-Phoxine-Safran (**D**) showing a substantially normal bone marrow (**F**) surrounded by two plasma cell infiltrates (**E** and **G**).

The conventional ^18^F-FDG-PET images were performed 1h PI and showed typical ^18^F-FDG distribution in brain, heart, muscles and intestines (Figure 4B). Except for skull lesions, the uptake correlated with the bioluminescence images for all lesions yet with limited uptake in the tumors (Figure 4, Figure 5 and Supplementary Table 1).

**Figure 6:**
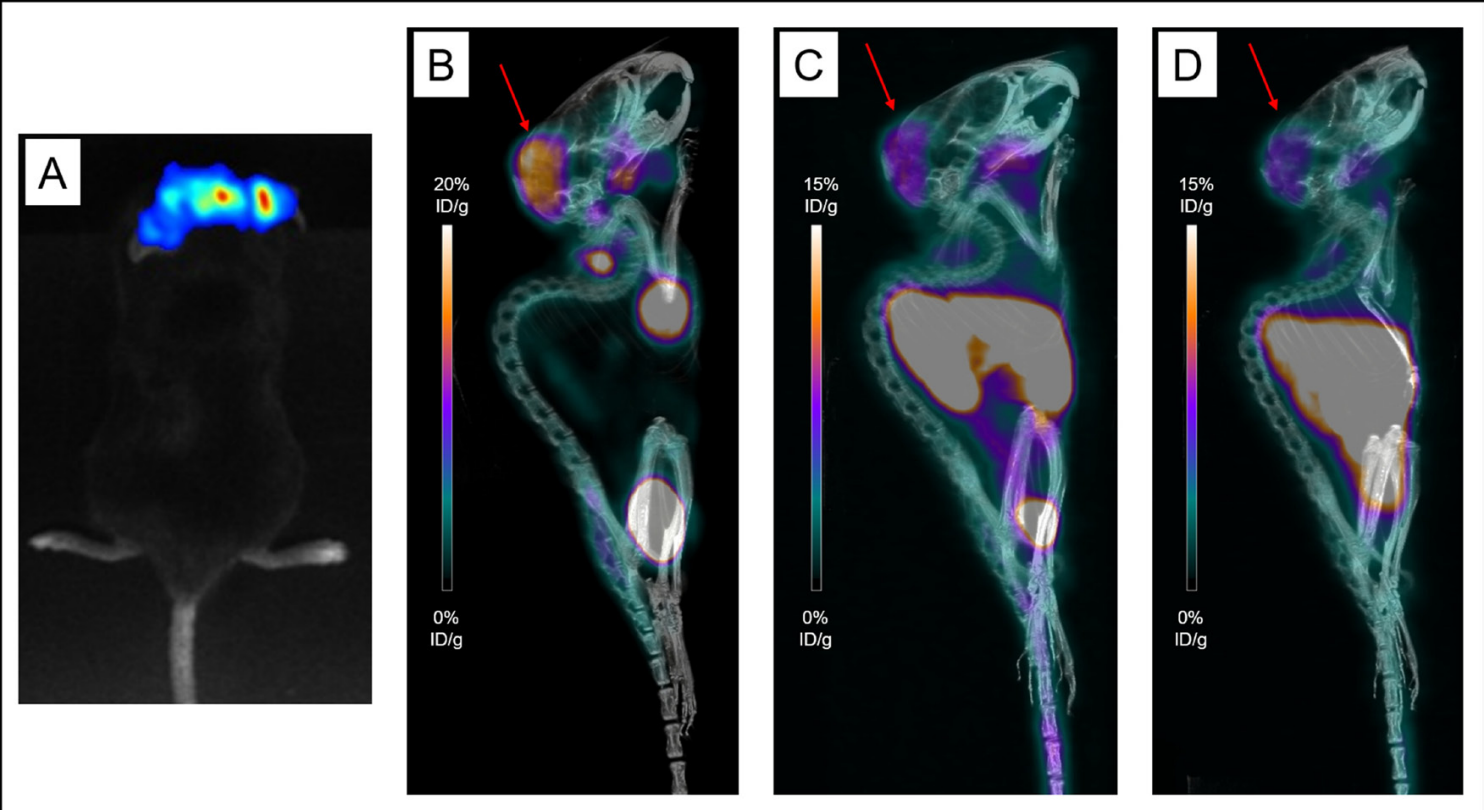
Various explorations conducted in Mouse 18 Bioluminescence imaging (**A**), maximum intensity projections of ^18^F-FDG-PET and CT imaging (**B**), maximum intensity projections of PET and CT imaging with ^64^CuCl_2_ at 2h post-injection (PI) (**C**) and 24 h PI (**D**) showing uptakes in the skull (Tumors are indicated by arrows).

PET imaging with ^64^Cu-TE2A-9E7.4 was performed 2h and 24h PI. Besides the physiological uptakes also observed in the SC model, bone and lymph node metastases were easily distinguished with excellent tumor-to-background ratios. Supplementary Table 1 shows the lesion territories for each imaging method. Imaging with ^64^Cu-TE2A-9E7.4 was able to detect all the lesions and lymph nodes observed with bioluminescence imaging except for the skull lesions of Mice 13, 14 and 15 and was also able to detect skull infringement of the Mouse 16 (Figure 4), undistinguishable on ^18^F-FDG-PET images. Higher contrast was observed at 24h PI compared to the images realized at 2 h PI.

**Figure 7:**
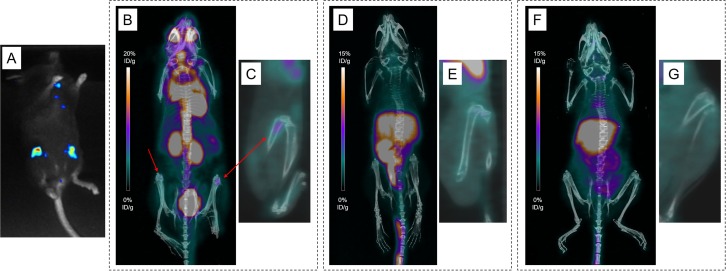
Various explorations conducted in Mouse 19 Bioluminescence imaging (**A**) and maximum intensity projections of ^18^F-FDG-PET and CT imaging (**B**), showing uptakes in both femurs (Tumors are indicated by arrows). No significative uptake seen on maximum intensity projections of PET and CT imaging with ^64^CuCl_2_ at 2 h post-injection (PI) (**D**) and 24 h PI (**F**). Representative sagittal images of the left femur on PET imaging (**C**, **E**, **G**).

PET imaging with ^64^CuCl_2_ was performed 2h and 24 h PI. Similarly to PET imaging in the SC tumor-bearing mice, high liver and kidney uptakes were also observed at all time points. For Mouse 18, skull infiltration was clearly visible at 2 h and 24 h PI yet with lesser contrast than on ^18^F-FDG-PET images (Figure 6). Besides, no tumor was individualized in Mouse 19 (Figure 7).

### Histology

The SC 5T33-Luc(+) tumors of Mice 1 and 7 and both femurs of Mouse 13 were collected for morphological analysis. Analysis of SC tumors showed numerous large and atypical plasma cells. Figure 5 presented the morphological analysis of the femurs of Mouse 13. A rich normal bone marrow was observed in the femur considered as negative on the PET images with ^64^Cu-TE2A-9E7.4. The femur considered as positive on PET images with ^64^Cu-TE2A-9E7.4 showed a substantially normal bone marrow surrounded by two cellular infiltrates with morphologies and characteristics similar to SC tumor’s analysis.

## DISCUSSION

Benefiting of major technological advances, molecular characterization of tumors has helped highlight biomarkers, useful in identifying cancer cells and understanding the variability of response to therapeutic agents. These biomarkers can also be used as targets and have thus enabled the development of more specific targeted therapies [15]. In medical practice, the identification of these biomarkers slowly but surely becomes a prerequisite before any treatment decision, leading to the concept of personalized medicine. Immuno-PET perfectly fits with this approach. Indeed, mAbs labeled with radionuclides represent promising probes for theranostic approaches, offering a non-invasive solution to assess *in vivo* target expression and distribution and to obtain reliable diagnostic, prognostic and therapeutic information [6]. MM appears to be a good candidate for this type of imaging. Although recent data from the literature suggest that ^18^F-FDG-PET is a promising technique for the assessment of MM with a strong prognostic value at diagnosis, developing a more specific radiopharmaceutical for this pathology seems relevant [16]. Besides, based on the evidence that copper is known to be critical for cell proliferation, angiogenesis, and tumor growth [17], and that increased copper ions were detected in cancer tissues [18], ^64^CuCl_2_ has been reported as a promising PET probe for imaging a variety of cancers [12–14, 19–22]. Interestingly, MM might again be a good candidate as a copper-chelator, ATN-224, was shown to induce programmed cell death in multiple myeloma cells emphasizing the essential role of the Copper ion in this pathology [23]. Our study demonstrates for the first time the efficiency of anti-CD138 immuno-PET and ^64^CuCl_2_ to detect MM lesions in a preclinical model.

The present work demonstrated the high targeted-tumor uptake of ^64^Cu-TE2A-9E7.4 in MM tumor-bearing mice. Based on our experience with the B-B4 mAb [24, 25], our group had developed this rat anti-mouse syndecan mAb (9E7.4, IgG2a κ isotype) that specifically recognizes the extracellular domain of mouse syndecan-1 [26]. This study showed that ^64^Cu-TE2A-9E7.4 binds effectively to CD138 tumors and allows MM imaging in a syngeneic mouse model with high contrast. Moreover, specificity of the observed *in vivo* tumor uptake was validated by the ^64^Cu-TE2A-IgG2ak Isotype control antibody biodistributions. With this latter, relative low tumor uptake, poor tumor-to-blood ratio and uniform distribution were observed likely due to the non-specific enhanced permeability and retention effect (EPR) [27, 28]. The biodistribution data with ^64^Cu-TE2A-9E7.4, in general, agreed well with the small animal PET quantification results and showed, as it is typical for radioimmunoconjugates, that the optimal time point for high-contrast imaging of tumors with ^64^Cu-TE2A-9E7.4 appears better at 24 h than 2 h PI. This corresponded to the time necessary for the distribution of the antibody to the targeted tissues and clearance of non-targeted tissues allowing for a satisfactory contrast. At 24 h PI, relative high uptakes of ^64^Cu-TE2A-9E7.4 was still found in several normal organs such as liver, spleen, kidneys and the digestive tract. These could be attributed *(i)* to hepatobiliary clearance of the radiotracer and its associated immune-complexes, *(ii)* the endogenous expression of CD138 by the microvilli of the basal membranes of hepatocytes and/or *(iii)* potential *in vivo* trans-chelation of ^64^Cu from TE2A by hepatic enzymes even if TE2A has previously demonstrated high *in vivo* kinetic stability as a chelator for ^64^Cu^2+^ ion. This liver uptake could greatly hamper the interpretation of PET images with ^64^Cu-TE2A-9E7.4 yet hepatic involvement is rare in multiple myeloma. In spite of this background distribution, ^64^Cu-TE2A-9E7.4 achieved higher resolutions’ contrasts than ^18^F-FDG-PET. Indeed, despite precautions taken in terms of anesthesia and warming to reduce cellular metabolism [29], ^18^F-FDG-PET showed significantly more non-specific bindings and persistence of a high background noise explained by residual metabolic radioactivity. This good performance is particularly emphasized by the results of the disseminated model. Indeed, in the literature, the widespread use of subcutaneous xenograft models usually allows for easy assessment of tumor burden. An important drawback to the use of syngeneic and orthotopic tumor model would be the signal observed from organs of clearance such as the liver and kidneys that may conceal disseminated tumors. Moreover, the use of a syngeneic model provides access to the true distribution of the vector, whether it is linked to the epitopic mapping on the whole animal or to the fixation due to the Fc fragment. Yet, in the present work, as shown in Figure 5, 64Cu-TE2A-9E7.4 images showed excellent tumor contrast for disseminated lesions’ detection. These data support ^64^Cu-anti-CD138 antibody as a promising imaging tool. As previously stated, CD138 is expressed at high levels in MM tumors and is a key regulator in the disease [7]. High levels of CD138 in patient serum are associated with poor prognosis in MM disease progression [30]. In the past several years, our group has proven anti-CD138 radioimmunotherapy (RIT) and α RIT to be effective in an immuno-competent preclinical MM model [24, 31]. Moreover, Phase I–II studies have been initiated with an antiCD138 mAb [9, 10]. Thus, a specific radiotracer targeting CD138 may have advantages for visualizing and monitoring the disease or as a companion diagnostic imaging tool available for selecting patients and predicting or monitoring response to CD138-targeted therapies.

In our work, ^64^Cu was chosen among the positron-emitting radioelements compatible with the biodistribution of a complete mAb. On the one hand, for its intrinsic resolution, better than Iodine-124, and on the other hand, with regard to biological behaviors and distributions of free dissociated radionuclides. Indeed, even if Zirconium-89 is a good candidate for labeled antibodies [32], uncomplexed Zirconium-89 localizes in the bone and could potentially represent a problem in the case of MM phenotypic imaging [33]. Moreover, several previous studies that have examined the use of antibodies coupled to ^64^Cu for PET with success [13, 19, 34]. Nevertheless, the main difficulty of this radionuclide is the need for stable chelation for binding the radioisotope to the carrier biomolecule [20, 35]. Biological media are in fact rich in metal cations, chelating proteins and reducing enzymes, competing with the chelator and causing dissociation of the complex copper-chelator. Futhermore, the relatively high tumor uptake of ^64^Cu in different types of human tumor tissue implies that use of unstable 64Cu-labeled PET tracers may result in non-specific tumor uptake [20]. Thus, in a recent study, Roosenburg et al have shown that depending on the type of chelator used for coupling the antibody with ^64^Cu, biodistribution profiles were not the same [34]. Strong absorption by the liver and other organs such as the intestines were reported because of the low *in vivo* stability of the various chelators. In our study, a DOTA derivative, TE2A was used as a chelating agent [36]. Its macrocyclic structure seems more suitable to the *in vivo* stabilization of copper. In this study, ^64^CuCl_2_ imaging was thus initially performed as a control of this potential copper release by the chelating agent.

Moreover, it has been recently demonstrated that ^64^Cu in its ionic form can directly be used as a probe for PET imaging of various types of cancers [13]. This study is the first report, to our knowledge, which explores the potential of ^64^CuCl_2_ for imaging in MM. The results of the biodistribution and PET quantification studies revealed significant uptake of ^64^CuCl_2_ in MM tumors. ^64^CuCl_2_ displayed rapid accumulation in the SC MM tumors which slightly decreased over time. ^64^CuCl_2_ has previously been reported to accumulate in various mouse models of cancers such as melanoma, colorectal cancer, prostate cancer, with heterogeneous uptake values or kinetics [12–14, 19–22]. Tumor uptake of ^64^CuCl_2_ is indeed expected to be variable based on the profile of various copper transporters, chaperons, and copper binding molecules and not simply depending on the expression level of hCtr1, supported by the findings from other investigators [14, 20]. With an uptake of 7.47 ± 2.52%ID/g at 2h PI, the performance of ^64^CuCl_2_ in our study looks similar to experiments in the mouse melanoma xenografts reported previously [12, 37]. Relative high uptake of ^64^CuCl_2_ was also observed in non-target organs such as liver, kidney, lung, gut and stomach. These results are in agreement with precedent findings of the literature [13]. Nevertheless, the performance of ^64^CuCl_2_ in the disseminated model finally showed its greatest limitation as it might not be suitable for detection of disseminated metastases. Indeed, even though most tissues (such as bones and brain) showed relatively low uptake of ^64^CuCl_2_, tumor-to-blood ratios and thus contrast remained poor with lower performances than ^18^F-FDG-PET. Thereby, albeit exploring copper metabolism as an imaging biomarker in MM seemed attractive, ^64^CuCl_2_ can’t directly be used as an effective probe for non-invasive visualization of tumors. All the opposite of ^64^Cu-TE2A-9E7.4. To further investigate the uptake of ^64^CuCl_2_ and compare it to ^64^Cu-TE2A-9E7.4 more completely, additional autoradiography studies were conducted in SC tumors. The digital autoradiography date showed a higher and significant accumulation with ^64^Cu-TE2A-9E7.4 than with ^64^CuCl_2_ confirming the specificity of this radioimmunoconjugate and its high uptake *in vivo*.

## MATERIALS AND METHODS

### Cell lines and cultures

The 5T33 murine MM cell line was kindly provided by Dr. Harvey Turner (Nuclear Medicine Service, Fremantle Hospital, Western Australia) with the permission of Dr. Jiri Radl (TNO Institute, Leiden, Netherlands) [38]. Cells were transfected with luciferase cDNA as previously described [25]. 5T33-Luc(+) were cultured in RPMI1640 medium (Gibco, Saint Aubin, France) containing 2 mM L-glutamine and 10% heat-inactivated fetal calf serum (PAA Laboratories / GE Healthcare Europe GmbH) at 37°C, 5% CO_2_, 95% humidity.

### 9E7.4 antibody

The 9E7.4 mAb was produced by immunization of a rat with a 40-amino-acid peptide (GeneCust, Luxembourg) derived from the murine CD138 protein (aa 90–130) (GenBank: CAA80254.1). Its characterization was ensured within the team as previously described [26]. The isotype of this antibody is IgG2a,κ, and its binding specificity was around 1 × 10^−10^ M.

### Rat IgG2ak isotype control

The Rat IgG2ak antibody (R&D Systems; ref 54447) was chosen as an isotype control. This latter is suitable for use as a negative control to assess non-specific binding of Rat IgG2ak antibodies to mouse cells.

### Animal model: subcutaneous tumor model and IV disseminated tumor model

Female C57BL/KalwRij mice were purchased from Envigo and housed under conventional conditions at the Experimental Therapeutic Unit animal facility (SFR François Bonamy, IRS-UN, University of Nantes, license number: B-44-278). Experiments were approved by the local veterinary committee (reference 00143.01) and carried out in accordance with relevant guidelines and regulations. Mice were 17 weeks old at the time of experiments.

Twelve mice were grafted subcutaneously (SC) with 2.10^6^ 5T33-Luc(+) cells suspended in 100 μL of PBS 20 days before the first PET images on both legs. Tumors were grown to a size of 0.3-0.8 cm in diameter.

MM has a high likelihood of disseminating to the bones. For the experimental disseminated model, 1.10^6^ 5T33-Luc(+) cells were suspended in 100 μL of PBS and injected via tail vein into 8 mice, 34 days before the first PET images. Mice were monitored for bone marrow lesions by bioluminescence imaging over 33 days.

### Bioluminescence imaging

Mice were serially imaged using bioluminescence imaging as previously described [24] to identify, to locate and to follow tumor progression.

The mice were anesthetized with intraperitoneal injection of 100 μL/10g of an anesthetic solution (consisting of 1 mL ketamine at 100 mg/mL (Panpharma); 0.5 mL xylazine at 20 mg/mL (Bayer); and 8.5 mL PBS). Mice were injected intraperitoneally with 100μL luciferin (Interchim, 12 mg/mL) 5 min before being imaged. Mice were imaged in ventral and dorsal positions using a Photon IMAGER ™ (Biospace Lab, Paris, France) for 30 seconds. The images were analyzed using the M3Vision ™ software (Biospace Lab, Paris, France).

### Labeling and controls with copper-64 (^64^Cu)

For *in vivo* experiments, three tracers with ^64^Cu were used: ^64^Cu-TE2A-9E7.4, ^64^Cu-TE2A-IgG2a k Isotype and ^64^CuCl_2_. Copper-64 (t_1/2_ = 12.7 h, β^+^; 17.8%, E_β + max_ = 656 keV, β^−^, 38.4%, E_β- max_ = 573 keV) was obtained from the ARRONAX cyclotron (GIP ARRONAX, Saint-Herblain, France) using the reaction ^64^Ni (p,n)^64^Cu and was delivered as ^64^CuCl_2_ in HCl 0.1N.

To radiolabel the 9E7.4 and the IgG2a k Isotype mAbs, the antibodies were previously modified using a copper chelating agent called TE2A-benzyl isothiocyanate according to the method previously described [36]. Briefly, mAbs were incubated in a solution of 0.1M of EDTA for 2 hours in order to chelate contaminant metals. EDTA was then removed and mAbs were concentrated at 4 mg/mL in carbonate buffer (0.3 M, pH 8.6) using a disposable Amicon Ultra-4 centrifugal unit (Millipore). For conjugation, the chelating agent TE2A-benzyl isothiocyanate was dissolved at 12 mg/mL and added to the mAbs at a ratio of 20 moles of TE2A to 1 mole of antibody. After an incubation overnight, the excess of TE2A was removed by a PD-10 disposable gel filtration column (GE Healthcare Life Science) eluted with 0.3 M ammonium acetate (pH 7) fractions of 500 μL. For radiolabeling, mAbs in 0.3 M ammonium acetate (pH 7) were incubated with a solution of 244 μL of ^64^CuCl_2_ (408 MBq, dissolved in HCl 0.1N) and 60 μL of 2.5 M ammonium acetate buffer (pH 7.0) for 20 minutes at 40°C. Then 10 μL of EDTA (pH 7; 10 mM) were added and a further incubation for 5 minutes at 40°C was performed. Radiochemical purity was determined by thin layer chromatography ITLC-SG, using a citrate buffer (pH 4.5; 0.1 M) and was 83%. The immuno-conjugate labeled with ^64^Cu was thus secondly purified by size exclusion chromatography using a PD-10 column (Sephadex G25, GE Healthcare). The radiochemical purity was finally assessed by ITLC-SG at 100%. At the end of the radiolabeling, the specific activity for ^64^Cu-TE2A-9E7.4 was 187 MBq/mg and the specific activity for ^64^Cu-TE2A-IgG2a k Isotype was 338.7 MBq/mg.

The immunoreactivity of ^64^Cu-TE2A-9E7.4 was determined using magnetic beads (Pierce, Thermo Scientific) labeled with a 40 amino acids peptide recognized by the 9E7.4 antibody according to the supplier’s protocol. One picomole of ^64^Cu-TE2A-9E7.4 was incubated 1 hour at room temperature with 20 μL of coated magnetic beads (10 mg/mL). Using a magnetic rack, supernatants containing non-reactive antibodies and magnetic beads were collected separately. The radioactivity in each fraction was measured in a gamma counter as previously described by Halime et al [36]. The radiolabeling yield and specific activity post-purification of the bioconjugate were 95 ± 2.8% and 188 ± 27 MBq mg^−1^ respectively and its immunoreactivity was 81 ± 7%.

### PET imaging

For the SC model, 3 mice were imaged with ^18^F-FDG-PET and ^64^Cu-TE2A-9E7.4 (Mice 1 to 3), 3 with ^18^F-FDG-PET and ^64^Cu-TE2A-IgG2a k Isotype (Mice 4 to 6) and 6 with ^18^F-FDG-PET and ^64^CuCl_2_ (Mice 7 to 12).

For the disseminated model, 5 mice were imaged with ^18^F-FDG-PET and ^64^Cu-TE2A-9E7.4 (Mice 13 to 17) and 3 mice with ^18^F-FDG-PET and ^64^CuCl_2_ (Mice 18 to 20).

For ^18^F-FDG-PET imaging, mice were fasted overnight (6h to 12h) with free access to water. Mice were warmed for at least one hour, anesthetized with inhaled isoflurane 5%, and intravenously injected with 10 MBq of ^18^F-FDG in a volume of 100 μL through the lateral tail vein. Mice were maintained under anesthesia for a 1h uptake period and then scanned (350–650 keV energy window, 20 min listmode acquisition, 3D rebinning followed by OSEM-MAP reconstruction) on a multi-modality preclinical imaging system (Inveon™, Siemens Healthcare). CT acquisitions (80 kV, 0.5 mA) were also performed immediately before the PET imaging. The reconstructed PET images were analyzed using Inveon Research Workplace (Siemens Healthcare). Manually drawn 3-dimensional volumes-of-interest (VOIs) were used to determine tissue uptake values on decay-corrected whole-body coronal images. By assuming a tissue density of 1 g/mL, the VOIs were converted to percentage of the injected radioactive dose per gram of tissue (% ID/g).

For ^64^Cu PET studies, similar procedures were followed 24h post-^18^F-FDG-PET imaging, except that no fasting was performed and imaging occurred at 2h and 24 h PI. Each mouse was intravenously injected with 10 MBq of radiotracer (^64^Cu-TE2A-9E7.4 or ^64^Cu-TE2A-IgG2a k Isotype or ^64^CuCl_2_) in a volume of 100 μL via the lateral tail vein. According to the ^64^Cu decay, the specific activity at the injection time was between 140 MBq/mg and 170 MBq/mg for ^64^Cu-TE2A-9E7.4 and between 320 MBq/mg and 335 MBq/mg for ^64^Cu-TE2A-IgG2a k Isotype.

### Biodistribution study

Tracer biodistribution studies were carried out in all the SC-tumor-bearing mice after PET imaging (*n* = 3 for each group): at 24 h PI for ^64^Cu-TE2A-9E7.4, at 24 h PI for ^64^Cu-TE2A-IgG2ak Isotype and at 2 h and 24 h PI for ^64^CuCl_2_. Tumor, blood, and other selected tissues (liver, kidney, gut, lungs, muscle, spleen, skin, brain, heart, flat bone, femur, and stomach) were dissected, weighed and counted on a calibrated and normalized gamma-counter. For each organ, the percentage of injected dose per gram (% ID / g) was calculated. The organ to blood ratios were also compared.

### Histology and digital autoradiography of tumors

SC tumors of Mice 1 and 7 were removed, fast-frozen in cold 2-methylbutane solution, embedded in optimal-cutting temperature compound, and cut into 10 μm sections using a cryomicrotome (CM3050 Leica Biosystems^®^). Sections were mounted on Superfrost^™^ slides and digital autoradiography images were obtained on a Beaver^®^ imager (Ai4R, Nantes, France). Image analysis was performed on the dedicated software Beamage^®^ (Ai4R, Nantes, France). Adjacent 10 μm slices were stained with hematoxylin-Phoxine-Safran and scanned using a slide-scanner Nanozoomer (Hamamatsu^®^).

Both femurs of Mouse 13 were collected, formalin-fixed and decalcified for 48 h (Decalc, Histolab). After paraffin-embedding, femurs were cut into 10 μm sections using a RM2255 microtome. Tissue sections were mounted on Superfrost^TM^ slides. Slices were stained with Hematoxylin-Phoxine-Safran by Cellular and Tissular Imaging Core Facility of Nantes University (MicroPICell) and scanned using a slide scanner, Nanozoomer Hamamatsu^®^.

### Statistical analysis

Statistical analysis was performed using GraphPad Prism version 7.00. Differences in uptake were tested for significance using the non-parametric Mann-Whitney test for two groups. Assessment of sensitivity and accuracy for ^64^Cu-TE2A-9E7.4 and ^18^F-FDG-PET in the disseminated disease model was done using bioluminescence and CT from the PET protocol as a reference. A *p* value below 0.05 was considered significant.

## CONCLUSIONS

The anti-CD138 antibody 9E7.4 conjugate to TE2A, radiolabeled with ^64^Cu, showed specific binding to CD138 *in vivo*. Immuno-PET data demonstrated that this radioimmunoconjugate can be used for noninvasive imaging of CD138 expression in MM and has high tumor-to-background tissue contrast, superior to ^18^F-FDG-PET. On the opposite, the potential of ^64^CuCl_2_ as a radiotracer for PET imaging of MM remains uncertain. These data support ^64^Cu-anti-CD138 antibody as a promising imaging tool for selecting patients before antibody-based therapy and RIT in particular. Future optimization studies will include testing CD38 which is expressed at lower levels in the liver than CD138, as a new target. Smaller vectors such as F(ab’)2 fragments could also offer faster biodistribution and present less catabolism by the liver.

## SUPPLEMENTARY MATERIALS FIGURES AND TABLES


