# Supplementary material for: Comparison of Immuno-PET of CD138 and PET imaging with 64CuCl2 and 18F-FDG in a preclinical syngeneic model of multiple myeloma
Source: Oncotarget. 2018 Jan 3;9(10):9061–72. doi: 10.18632/oncotarget.23886 (PMC5823645; doi:10.18632/oncotarget.23886)
Supplement: Supplementary file 1 [file oncotarget-09-9061-s001.pdf]

Comparison of Immuno-PET of CD138 and PET imaging with $^{64}CuCl_2$ and ^{18}F-FDG in a preclinical syngeneic model of multiple myeloma

SUPPLEMENTARY MATERIALS

Supplementary Table 1: Different PET imaging conducted in mice and lesions visualized on the different imaging methods

	Bioluminescence imaging	^{18}F-FDG-PET	^{64}CuCl2	^{64}Cu-TE2A-9E7.4
Subcutaneous tumor model				
Mouse 1		Sc x 2		Sc x 2
Mouse 2		Sc x 2, Ln		Sc x 2, Ln
Mouse 3		Sc x 2		Sc x 2
Mouse 4		Sc x 2		
Mouse 5		Sc x 2		
Mouse 6		Sc x 2		
Mouse 7		Sc x 2	Sc x 2	
Mouse 8		Sc x 2	Sc x 2	
Mouse 9		Sc x 2	Sc x 2	
Mouse 10		Sc x 2	Sc x 2	
Mouse 11		Sc x 2	Sc x 2	
Mouse 12		Sc x 2	Sc x 2	
IV disseminated tumor model				
Mouse 13	Sk, Sa, Mb	Sa, Mb		Sa, Mb
Mouse 14	Sk	0		0
Mouse 15	Sk, Sa, Ln	Sa, Ln		Sa, Ln
Mouse 16	Sk, Sa, Iw	Sa, Iw		Sk, Sa, Iw
Mouse 17	Sk, Sa, Mb	Sk, Sa, Mb		Sk, Sa, Mb
Mouse 18	Sk	Sk	Sk	
Mouse 19	St, Mb	St, Mb	-	
Mouse 20	Sk, Sp	Sk, Sp	Sk	

Lesional territories: Sc: subcutaneous; Ln: lymph node; Sk: skull; Sa: Sacrum, Iw: iliac wing; Mb: member; St: sternum, Sp: spine.
